# The Transcription Factor IRF6 Co-Represses PPARγ-Mediated Cytoprotection in Ischemic Cerebrovascular Endothelial Cells

**DOI:** 10.1038/s41598-017-02095-3

**Published:** 2017-05-19

**Authors:** Rongzhong Huang, Zicheng Hu, Yuxing Feng, Lehua Yu, Xingsheng Li

**Affiliations:** 1 Department of Rehabilitation Medicine, the Second Affiliated Hospital of Chongqing Medical University, Chongqing, China; 2 Department of Neurology, Institute of Surgery Research, Daping Hospital, Third Military Medical University, Chongqing, China; 3 Department of Neurology, the Ninth People’s Hospital of Chongqing, Chongqing, China; 4 Department of Gerontology, the Second Affiliated Hospital of Chongqing Medical University, Chongqing, China

## Abstract

Activation of peroxisome proliferator-activated receptor gamma (PPARγ) in the cerebrovascular endothelium is a key suppressor of post-stroke brain damage. However, the role of PPARγ’s co-regulators during cerebral ischemia remains largely unknown. Here, we show that the transcription factor IRF6 is a novel PPARγ co-regulator that directly binds to and suppresses PPARγ activity in murine cerebrovascular endothelial cells. Moreover, IRF6 was also revealed to be a transcriptional target of PPARγ suppression, with PPARγ silencing significantly promoting IRF6 expression in cerebrovascular endothelial cells. In addition, IRF6 silencing significantly promoted pioglitazone’s cytoprotective effects in ischemic murine cerebrovascular endothelial cells. Mechanistically, IRF6 significantly suppressed PPARγ’s transcriptional inhibition of the ischemia-induced, pro-apoptotic microRNA miR-106a. In conclusion, we identified IRF6 as a novel PPARγ co-suppressor that serves a key role in suppressing PPARγ-mediated cerebrovascular endothelial cytoprotection following ischemia. Further investigation into IRF6 and other PPARγ co-regulators should provide additional insights into PPARγ’s cytoprotective role in the cerebrovascular endothelium following stroke.

## Introduction

Stroke is currently the second-leading cause of mortality worldwide^1^. Moreover, stroke-related deaths (5.9 million deaths) as well as lost disability-adjusted life-years (102 million life-years) have both significantly increased since 1990 (26% increase and 12% increase, respectively)^1^. Unfortunately, aside from endovascular thrombectomy and thrombolysis in the setting of an acute stroke, there are few effective neuroprotective therapies for stroke^2^. Thus, a better understanding of the cerebrovascular pathophysiology underlying stroke’s adverse effects is still needed.

To this end, the transcription factor peroxisome proliferator-activated receptor-gamma (PPARγ) appears to be a promising therapeutic target, as endothelial PPARγ has shown anti-inflammatory, anti-thrombotic, and anti-atherosclerotic effects^3, 4^. In animal models of stroke, PPARγ agonists – such as thiazolidinediones and fibrates–have been shown to alleviate post-stroke brain damage^5, 6^. Moreover, thiazolidinediones show evidence of reducing recurrent stroke events in patients with stroke or transient ischemic attacks (TIAs)^7^. Mechanistically, PPARγ target gene transcription is normally repressed by co-repressors; however, upon ligand binding, PPARγ changes its conformation and recruits co-activators to promote target gene transcription^8^. Therefore, PPARγ’s end-effects depend largely on its network of co-regulators^8^.

Recent research by Yin *et al*. has identified several co-regulators of PPARγ that mediate PPARγ’s effects in cerebrovascular endothelial cells^9^. One such co-regulator–interferon regulatory factor 6 (IRF6) – has been shown to play a key role in epidermal differentiation and craniofacial development^10^; specifically, human IRF6 mutations have been conclusively linked with congenital cleft lip/palate disorders^11^. Accordingly, IRF6-null mice display hyperproliferative epidermal tissue as well as craniofacial abnormalities^12^. As the bulk of IRF6 research has focused on epidermal differentiation and craniofacial development^13, 14^, IRF6’s role as a PPARγ co-regulator in ischemic cerebrovascular disease (if any) remains unknown.

In the current study, we characterize IRF6’s role as a novel PPARγ co-repressor in the ischemic cerebrovascular endothelial cells. The current findings should aid future researchers in better characterizing IRF6 as a novel therapeutic target for modulating PPARγ-associated signaling cascades for purposes of stroke treatment.

## Materials and Methods

### Ethics Statement

The animal protocols were approved by the Animal Care and Use Committee the second affiliated hospital of Chongqing Medical University. All animal procedures were conducted in accordance with the Guide for the Care and Use of Laboratory Animals (National Institutes of Health, Bethesda, MD, USA). All efforts were made to minimize animal discomfort and suffering.

### Initial Screening for IRF6-PPARγ Co-Regulation

A co-regulator system was applied to screen for IRF6-PPARγ co-regulation as previously described by Yin *et al*. with minor modifications^9^. Briefly, a Gateway cloning system was used to construct a Gal4-IRF6 fusion plasmid through fusing the IRF6 open reading frame (ORF) to the Gal4-DNA-binding domain vector. AD293 cells were seeded onto 96-well plates, and Lipofectamine 2000 (Invitrogen, Carlsbad, CA, USA) was used to transfect the AD293 cells with a UAS-luciferase reporter (10 ng) as well as the Gal4-IRF6 fusion plasmid (20 ng) with or without pcDNA3.1 Flag-PPARγ (100 ng). Forty-eight hours post-transfection, *in vitro* luciferase activity was assessed.

### Mice Subjects

Wild-type (WT) C57BL/6 J male mice (n = 24, 8–10 weeks old) were purchased for this study. Mice were housed in individual cages with a 12 hour/12 hour light/dark cycle at 23 °C and 45% humidity. Mice were offered standard chow and water ad libitum. At the time of experimentation, all mice subjects were of similar weight and age and were randomly allocated to experimental groups.

### Adenoviral-Mediated IRF6 Knockdown in Mice Subjects

As previously described by Korbelin *et al*.^15^, here we employed the NRGTEWD peptide-displaying adeno-associated virus vector (termed AAV-BR1) that has demonstrated the strongest specificity for murine brain endothelial cells. The most efficacious murine *Irf6* shRNA clone TRCN0000085329, as previously reported by Ke *et al*.^16^, was cloned into the pAAV-CAG-2A-eGFP plasmid, generating a pAAV-CAG-shIRF6-2A-eGFP plasmid.

We produced the recombinant AAV vectors via triplicate transfection of HEK293T cells as previously described by Korbelin *et al*.^15^. Briefly, HEK293T cells were cultured in DMEM with 10% fetal calf serum plus 1% penicillin/streptomycin (Invitrogen) at 37 °C and 5% CO_2_ and then transfected with plasmid DNA via linear polyethylenimine (Polysciences, Milwaukee, WI, USA). The following plasmids were employed for transfection: the pAAV-CAG-shIRF6-2A-eGFP plasmid (described above), a pXX6 helper plasmid, and a pXX2-187 plasmid for the murine brain endothelial cell-specific AAV-BR1 capsid. Three days post-transfection, HEK293T cells were lysed in PBS-MK by freeze-thaw cycling. The AAV-BR1 vectors were then purified via affinity column (HiTrap column with AVB Sepharose High Performance resin, GE Healthcare Life Sciences, Pittsburgh, PA, USA). To quantify the vector stocks, quantitative RT-PCR was employed to measure genomic titers with the following CAG-specific primers: forward, 5′-GGA CTC TGC ACC ATA ACA CAC-3′ and reverse, 5′-GTA GGA AAG TCC CAT AAG GTC A-3′.

Under 2.5% isoflurane anesthesia, recombinant AAV-BR1 vectors (5.0 × 10^10^ genomic particles per subject) were injected into the mice subjects via the tail vein. Cerebral microvessels were isolated from the cortical brain tissue for experimentation fourteen days following vector administration. In order to validate recombinant AAV-BR1 vector transmission, total DNA from cerebral microvessels was extracted via a DNeasy Tissue Kit (Qiagen, Valencia, CA, USA) and a Precellys homogenizer with ceramic beads (Peqlab, Erlangen, Germany) and then quantified via spectral photometry (ND-1000, NanoDrop Technologies, Wilmington, DE, USA). Recombinant AAV-BR1 vector DNA from the cerebral microvessels was measured via quantitative RT-PCR via the CAG-specific primers listed above. Vector copy counts were normalized to total DNA content. In addition, knockdown of IRF6 was validated by Western blotting as described below (Supplementary Figure 1).

### Murine Model of Transient Cerebral Ischemia

Yin *et al*.’s model of transient focal cerebral ischemia was constructed by middle cerebral artery (MCA) occlusion^17^. During the entire procedure, regional cerebral blood flow, mean arterial pressure, heart rate, arterial blood gases, and rectal temperature were monitored, and the rectal temperature was maintained at 37.0 ± 0.5 °C using a feedback-controlled heating pad (SS20-2, Huaibei Zhenghua Bioinstrumentation Equipment Ltd., Anhui, China). Briefly, the mice subjects were first anaesthetized with ketamine (100 mg/kg) and xylazine (10 mg/kg). A midline skin incision was performed to expose the left common carotid artery, followed by electrocoagulation of the arterial branches. A 6-0 rounded tip nylon suture (length: 2 cm) was inserted into the external carotid artery and advanced into the internal carotid artery until regional cerebral blood flow was lowered to ~15% of the baseline level. After 30 minutes of MCA occlusion, regional cerebral blood flow was returned via suture removal. Sham controls underwent identical operative procedures without occluding the MCA. After the 30-minute post-occlusion resting period, the mice subjects were left alone to recover for a 24-hour period in an air-ventilated incubator (24.0 ± 0.5 °C) immediately prior to sacrifice. For the pioglitazone-based experiments, pioglitazone (2 mg/kg) was i.p. injected at the beginning of the 24-hour recovery period. Following sacrifice, brains were quickly excised for further experimentation.

### Cerebral Microvessel Isolation

We isolated the cerebral microvessels from the cortical brain tissue as previously described by Yin *et al*.^18^. Briefly, the mice subjects were sacrificed by decapitation after anesthesia. Whole cerebral cortices were immediately dissected out and immersed in ice-cold buffer A (103 mM NaCl, 15 mM Hepes, 4.7 mM KCl, 2.5 mM CaCl_2_, 1.2 mM MgSO_4_, 1.2 mM KH_2_PO_4_, pH 7.4). The whole cortices were homogenized in a 5x volume excess of buffer B (103 mM NaCl, 25 mM HCO_3_, 15 mM Hepes, 10 mM glucose, 4.7 mM KCl, 2.5 mM CaCl_2_, 1.2 mM MgSO_4_, 1.2 mM KH_2_PO_4_, 1 mM sodium pyruvate, and 1.0 g/100 ml dextran, pH 7.4), and the resulting homogenate was suspended in a matching volume of 25% BSA. The suspension was centrifuged for 30 min at 5800 g (4 °C). The resulting pellet was recovered in buffer B and spun down for 5 min at 500 g. The vessel pellet was frozen down to −80 °C until further analysis.

### Primary Culture of Murine Cerebrovascular Endothelial Cells

Murine cerebrovascular endothelial cells were cultured from the mice subjects as previously described by Yin *et al*.^9^. Following sacrifice, cerebral cortices were dissected out, homogenized, and filtered. The filtered homogenate was then digested with collagenase B followed by collagenase/dispase (Roche Diagnostics, Indianapolis, IN, USA). The digested homogenate was then centrifuged in a 40% Percoll solution. The second band (which contains the cerebral microvasculature) was extracted by pipette and plated on collagen-coated dishes. The cerebrovascular endothelial cells were cultured to ~90% confluency (4–6 passages) prior to experimentation.

### Co-Immunoprecipitation

Co-immunoprecipitation was performed as previously described by Yin *et al*. with minor modifications^9^. Briefly, 1 × lysis buffer (Promega, Madison, WI, USA) was used to lyse the cerebrovascular endothelial cells. The lysate was centrifuged down for 15 min at 12000 g (4 °C). Protein G-PLUS agarose (Santa Cruz Biotechnology, Santa Cruz, CA, USA) was used to pre-clear the supernatants for one hour (4 °C), which was followed by overnight incubation (4 °C) with an anti-PPARγ antibody (1:200; catalog # sc-7273, Santa Cruz Biotechnology) or an anti-IRF6 antibody (1:500; catalog # NBP1-51911, Novus, Littleton, CO, USA). Normal IgG was applied as the negative control. Protein G-PLUS agarose was used to pull down the immunocomplexes for 1 hour (4 °C), followed by washing thrice with washing buffer. To exclude nucleic acid-mediated effects, co-immunoprecipitation was also performed in the presence of benzonase (10 units per reaction) for 30 minutes. Sodium doecyl sulphate polyacrylamide electrophoresis (SDS-PAGE) was applied for separation, followed by Western blotting with the anti-PPARγ antibody (Santa Cruz Biotechnology) or the anti-IRF6 antibody (Novus) as described below.

### Adenoviral Transfection for PPARγ and IRF6 Gain-of-Function or Loss-of-Function

The cerebrovascular endothelial cells were transfected with an adenoviral vector as previously described by Yin *et al*. with minor modifications^9^. Briefly, the murine *Pparg* gene sequence or the murine *Irf6* gene sequence was amplified followed by cloning into the adenoviral pCMVTrack vector. For constructing the adenoviral vector delivering the small hairpin PPARγ (PPARγ shRNA) gene in order to silence PPARγ expression, either the murine *Pparg* shRNA clone TRCN0000001672^19^ or a random, non-binding 29-mer small hairpin green fluorescent protein (GFP) sequence was cloned into the adenoviral vector. Similarly, for constructing the adenoviral vector delivering the small hairpin IRF6 (IRF6 shRNA) gene in order to silence IRF6 expression, either the murine *Irf6* shRNA clone TRCN0000085329^16^ or a random, non-binding 29-mer small hairpin GFP sequence was cloned into the adenoviral vector.

The foregoing plasmids were co-transformed along with the AdEasy vector into *Escherichia coli*. The resulting clones were isolated, digested, and transfected into HEK293 cells for amplification. For 10 days, the transfected HEK293 cells were scanned for GFP expression and were finally collected for viral purification via a CsCl gradient. The resulting adenoviral vector was applied to infect the cerebrovascular endothelial cells for 48 hours. Then, overexpression and silencing of PPARγ and IRF6 were validated by Western blotting as described below (Supplementary Figure 2). An adenoviral vector with a GFP gene was used as a control^17^. Only populations of transfected cerebrovascular endothelial cells displaying greater than 70% GFP + cells were kept for later experimentation.

### Western Blotting

Murine cerebrovascular endothelial cells underwent Western blotting as previously described by Yin *et al*.^9^. Briefly, the cells were lysed via lysis buffer to isolate the total protein. Equal protein amounts were subjected to SDS-PAGE followed by transfer to polyvinyldifluoride (PVDF) membranes. PVDF membranes were then blocked in a blocking solution (5% non-fat milk in Tris-buffered saline) followed by incubation with the following primary antibodies for two hours at room temperature: anti-PPARγ (1:200; catalog # sc-7273, Santa Cruz Biotechnology), anti-CD36 (1:200; catalog # sc-9154, Santa Cruz Biotechnology), and β-actin (1:500; catalog # sc-47778, Santa Cruz Biotechnology). The membranes were then incubated for one hour at room temperature with the appropriate alkaline phosphatase-conjugated secondary antibodies (Promega). The ProtoBlot AP System (Promega) was applied to assess the color reaction according to the kit’s instructions.

### Luciferase Reporter Assays

Luciferase reporter assays in the cerebrovascular endothelial cell cultures were conducted as previously described by Yin *et al*. with minor modifications^9^. Prior to performing the assays, the cerebrovascular endothelial cells were cultured in 24-well plates to ~50% confluence.

For analyzing IRF6 promoter-driven transcriptional activity, the wild-type murine IRF6 gene promoter was PCR-amplified from the murine genome, which was cloned into the pGL 4.10 Luciferase vector (Promega). A bioinformatics analysis revealed two putative PPAR binding sites (PPREs) in the promoter segment: −2206/−2190 bp (IRF6 PPRE site 1 WT) and −11626/−11606 bp (IRF6 PPRE site 2 WT). Therefore, using a QuikChange XL Site-Directed Mutagenesis Kit (Stratagene, La Jolla, CA, USA), two mutant IRF6 promoters (PPRE site 1 mutant and PPRE site 2 mutant) were constructed by substituting four bp from each of the two IRF6 WT PPRE sites as follows (mutations indicated by underline): the IRF6 PPRE site 1 mutant was 5′-ATC CAG GTA CC GAG TGA-3′, and the IRF6 PPRE site 2 mutant was 5′-GTC AGG GTC CCC TGC AGA GTG-3′. The IRF6 WT and mutant constructs were validated by sequencing. Using Lipofectamine 2000 (Invitrogen), the three IRF6 promoter plasmids (i.e., IRF6 PPRE WT, IRF6 PPRE site 1 mutant, and IRF6 PPRE site 2 mutant) were each co-transfected along with a Renilla luciferase reporter vector (pRL-TK) into cerebrovascular endothelial cells for four hours. The cells were then shifted to the standard growth medium for an additional 48 hours. For pioglitazone-based experiments, the cerebrovascular endothelial cells were incubated overnight with pioglitazone (10 µM) in the presence or absence of adenovirally-transfected PPARγ or GFP. A luminometer (Model GD-1, Ruike, Xi’an, China) was used to measure luciferase activity with a dual luciferase assay kit. Each transfection procedure was performed at least three times in triplicate.

For analyzing miR-106a promoter-driven transcriptional activity, using Lipofectamine 2000 (Invitrogen), cerebrovascular endothelial cells were co-transfected with a plasmid vector carrying either (i) the luciferase gene under the control of three tandem PPAR response elements (PPRE) (i.e., PPRE × 3 TK-luciferase) or (ii) a pGL 4.10 luciferase vector with a 1.9-kb fragment of the microRNA-106a (miR-106a) promoter containing a WT or mutated PPRE binding site at −2262/−2248 bp (four bp mutation indicated by underline: GTA GGG ACC CGG TCA). Transfection with the pRL-TK vector was applied as an internal control. Post-transfection, cerebrovascular endothelial cells were cultured in Opti-MEM I for four hours and were then infected with an adenovirus delivering PPARγ, IRF6, or GFP for an additional 48 hours. A luminometer (Model GD-1, Ruike, Xi’an, China) was applied to measure luciferase activity with a dual luciferase assay kit.

### Oxygen-Glucose Deprivation (OGD) Exposure

In order to mimic *in vivo* ischemia, the cerebrovascular endothelial cells were exposed to OGD conditions as previously described by Yin *et al*. with minor modifications^9^. Briefly, confluent cerebrovascular endothelial cells were placed in a deoxygenated glucose-free Hanks’ Balanced Salt Solution and incubated in a temperature-regulated anaerobic chamber for 16 hours (37.0 ± 1.0 °C; (Shanghai CIMO Medical Instrument Co., Shanghai, China) that contained a an anaerobic gas mixture (95% N_2_, 5% CO_2_). Non-OGD cells were maintained under normoxic conditions. For pioglitazone-based experiments, the cerebrovascular endothelial cells were incubated overnight with pioglitazone (10 µM) in the presence or absence of adenovirally-transfected PPARγ, PPARγ-shRNA, IRF6, or GFP before OGD.

### Transfection with miR-106a Mimic and Inhibitor

Murine cerebrovascular endothelial cells were cultured in 24-well plates. Using Lipofectamine 2000 (Invitrogen), the cells were transfected with a miR-106a mimic, miR-106a inhibitor, or a scrambled-sequence microRNA negative control (all at a 50 nM final concentration; miRIDIAN mimic, Dharmacon, Lafayette, CO, USA). The cells then underwent OGD exposure as described above prior to harvesting.

### Cell Viability Assays

Cell viability in the murine cerebrovascular endothelial cells was assessed as previously described by Yin *et al*. with minor modifications^9^. Briefly, cell viability levels were analyzed through an MTT assay (MTT Cell Proliferation and Cytotoxicity Assay Kit, Beyotime Institute of Biotechnology, Nanjing, China) and a lactate dehydrogenase (LDH) assay (LDH Activity Assay Kit, Jiancheng Co., Nanjing, China).

### Caspase-3 Activity and DNA Fragmentation Assays

Caspase-3 activity levels in isolated cerebral microvessels as well as murine cerebrovascular endothelial cells were assayed as previously described by Yin *et al*. with minor modifications^17^. Briefly, 1 × lysis buffer was applied to lyse cerebrovascular endothelial cells. Following 10 min on ice, the cell lysate was spun down for 10 min at 10000 g (4 °C). The resulting supernatant was subjected to a caspase-3 activity assay according to the kit’s instructions (Caspase-3 Assay Kit, Beyotime Institute of Biotechnology). A microtiter plate reader was used to read optical density (OD) values at 405 nm. Relative caspase-3 activity was calculated by comparing OD levels from the experimental groups against those of controls.

DNA fragmentation levels in isolated cerebral microvessels were assayed as previously described by Yin *et al*.^17^. Briefly, we first extracted the genomic DNA from isolated cerebral microvessels. Then, a commercial apoptotic DNA-ladder kit (Roche) was employed to measure DNA fragmentation levels.

### Evans Blue Extravasation Assay

Evans Blue extravasation was assayed as previously described by Yin *et al*.^17^. Briefly, 23 hours following MCA occlusion, mice subjects were injected via tail vein with 4% Evans Blue (100 ml). After one hour, mice subjects were PBS-perfused, and their brains were immediately excised. The two brain hemispheres were separated, and each brain hemisphere was homogenized in N, N-dimethylformamide. The homogenates were then centrifuged for 45 min at 25000 g, and the resulting supernatants were collected. Evans Blue extravasation was calculated as follows: (A620 nm [(A740 nm + A500 nm) ÷ 2])/mg wet weight. Baseline Evans Blue levels (as measured from the non-ischemic brain hemisphere) were subtracted from those of the matching contralateral ischemic brain hemisphere.

### Quantitative Real-Time Reverse Transcriptase Polymerase Chain Reaction (qRT-PCR)

qRT-PCR was performed with an iScript SYBR Green RT-PCR Kit and an iCycler iQ5 RT-PCR thermocycler (both Bio-Rad, Hercules, CA, USA) as previously described by Yin *et al*. with minor modifications^9^. The IRF6 primer sequences applied were as follows: forward, 5′-CAT GCC ATT TAT GCC ATC AG-3′ and reverse, 5′-AAA AGG CGG CTG CTT CTC TA-3′. mRNA expression was normalized to 18 S RNA levels.

### TaqMan Assay for miR-106a Expression

miR-106a expression in the murine cerebrovascular endothelial cells or the isolated cerebral vessels was assayed as previously described by Yin *et al*. with minor modifications^17^. Briefly, a miRNeasy Mini Kit (Qiagen, Valencia, CA, USA) was used to isolate total RNA (10 ng) was isolated from the murine cerebrovascular endothelial cells or the isolated cerebral vessels, which was then reverse transcribed with a TaqMan MiRNA Reverse Transcription Kit (Applied Biosystems, Foster City, CA, USA) using specific miRNA reverse transcriptase primers, dNTPs (with dTTP) (100 mM), reverse transcriptase (50 U), and RNase inhibitor (0.4 U) for 30 mins at 16 °C, for 30 mins at 42 °C, and for 5 mins at 85 °C. Then, a TaqMan MiRNA Assay Kit (Applied Biosystems) was used to perform PCR reactions for 10 mins at 95 °C, followed by 40 cycles of 15 sec at 95 °C and 60 sec at 60 °C. Each PCR reaction (20 μl) consisted of the RT reaction product (1.33 μl), TaqMan 2x Universal PCR Master Mix (10 μl), and 20 × TaqMan MicroRNA Assay reagent for miR-106a (1 μl). Relative miR-106a expression was normalized to snoRNA202 expression.

### Chromatin Immunoprecipitation Assay

A chromatin immunoprecipitation (ChIP) analysis of IRF6’s effect upon PPARγ binding to the miR-106a promoter was performed in murine cerebrovascular endothelial cells as previously described by Yin *et al*. with minor modifications^17^. Briefly, murine cerebrovascular endothelial cells were infected with an adenovirus delivering PPARγ, IRF6, PPARγ + IRF6, or GFP for 48 hours. Following fixation, a chromatin immunoprecipitation (ChIP) assay was conducted with an EZ-ChIP assay kit (Millipore). Total DNA extracted from murine cerebrovascular endothelial cells was sonicated into 0.5–1 kb fragments as assessed by ethidium bromide gel electrophoresis. The purified sheared chromatin and magnetic beads were then immunoprecipitated with a specific anti-PPARγ antibody or normal rabbit IgG (Santa Cruz Biotechnology) on a rotator at 4 °C. The chromatin-antibody-magnetic bead complexes were rinsed with low-salt, high-salt, LiCl, and TE buffers. The chromatin complexes were eluted and incubated for two hours at 62 °C. The eluted DNA fragments were then purified for PCR-based amplification. Employing bioinformatics to analyze the murine miR-106a promoter, we identified a putative PPRE site at −2262/−2248 bp upstream of the start codon and then PCR-amplified the region containing this putative PPRE site. As a negative control, no discernable DNA binding was observed in IgG immunoprecipitates.

### Statistical Analysis

Data were reported as means and associated standard errors of the mean (SEMs) from triplicate samples derived from three independent experiments. Pairwise comparisons were analyzed via two-tailed Student’s *t*-testing, while other comparisons were analyzed via one-way analysis of variance (ANOVA) followed by Bonferroni’s post-hoc testing. A *P*-value of less than 0.05 was deemed statistically significant.

## Results

### **I**RF6 Binding to PPARγ Inhibits PPARγ Activity in Cerebrovascular Endothelial Cells

**Figure 1:**
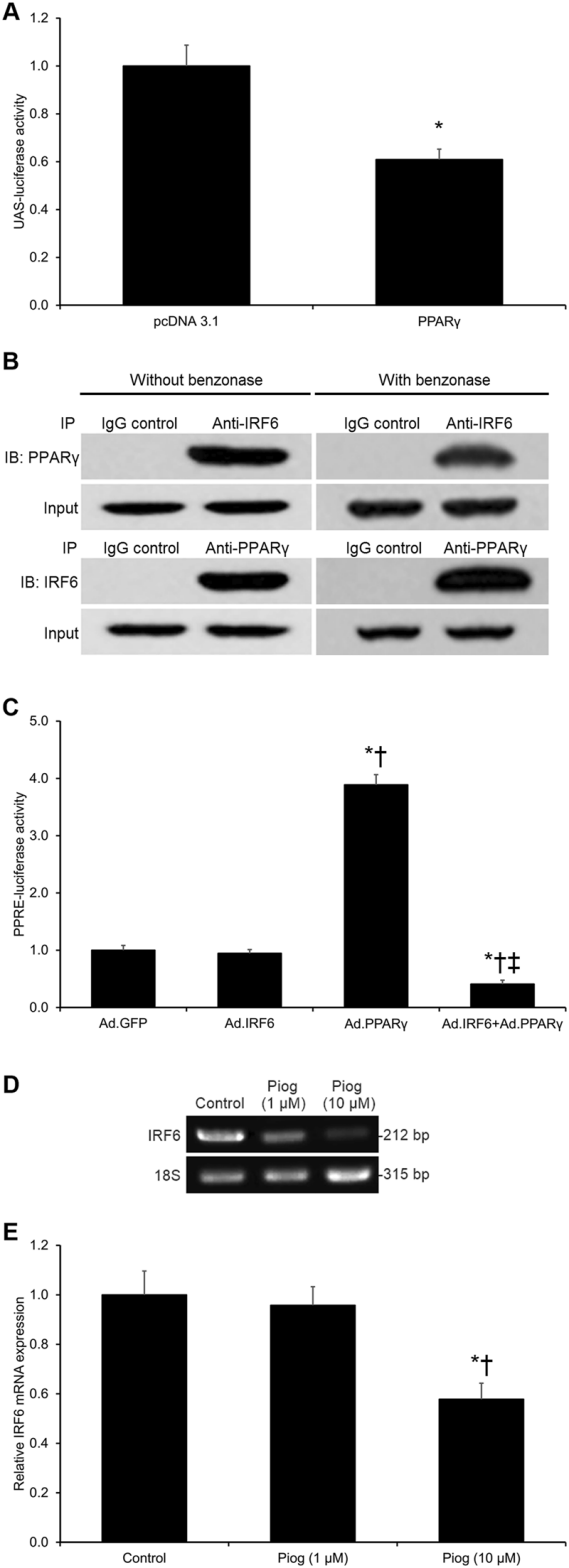
IRF6 Identified as a PPARγ Co-Regulator in Cerebrovascular Endothelial Cells. (**A**) A luciferase reporter assay was performed by co-transfecting HEK293 cells with UAS-Luc, PPARγ, and IRF6-Gal4-DBD vectors. *P < 0.05 versus pcDNA 3.1 group. (**B**) Co-immunoprecipitation (IP) assays on the total protein content from murine cerebrovascular endothelial cells revealed that IRF6 directly binds to PPARγ. Treatment with benzonase did not significantly affect IRF6-PPARγ binding. Cropped blots are displayed here. (**C**) PPRE transcriptional activity was assayed in murine cerebrovascular endothelial cells co-transfected with an adenoviral IRF6 vector and the 3 × PPRE-Luc vector in the presence of an adenoviral PPARγ vector or a GFP vector. Although IRF6 did not significantly affect PPRE activity at basal levels, IRF6 did significantly inhibit PPARγ-driven PPRE transcriptional activity. *P < 0.05 versus Ad.GFP group, ^†^ *P* < 0.05 versus Ad.IRF6 group, ^‡^ *P* < 0.05 versus Ad.PPARγ group. (**D,E**) Real-time PCR on the total RNA content from murine cerebrovascular endothelial cells treated with pioglitazone revealed that PPARγ activation by pioglitazone (10 µM) significantly inhibits IRF6 mRNA expression. Cropped blots are displayed here. *P < 0.05 versus Control group, ^†^ *P* < 0.05 versus Pioglitazone (1 µM) group. Data are reported as means ± standard errors of the mean (SEMs).

To investigate Yin *et al*.’s previous hypothesis that IRF6 may be a novel co-regulator of PPARγ^9^, the transcription factor IRF6 was chosen as the focus of this study. First, applying a co-regulator reporter assay, we found that IRF6’s transcriptional activity was significantly inhibited by PPARγ (*p* < 0.05, Fig. 1A). A co-immunoprecipitation experiment was conducted to validate the physical interaction between IRF6 and PPARγ. We found that IRF6 directly binds to PPARγ in a DNA-independent manner (Fig. 1B), which suggests that IRF6 co-regulates PPARγ. In order to test this hypothesis, a PPRE-luciferase activity assay was conducted in murine cerebrovascular endothelial cells to assess IRF6’s effect upon the transcriptional activity of PPARγ. We found that IRF6 significantly inhibits PPRE activity under conditions of adenovirus-driven PPARγ overexpression, but IRF6 did not significantly affect PPRE activity at basal PPARγ expression levels (*p* < 0.05, Fig. 1C). Moreover, pioglitazone-driven PPARγ activation significantly inhibited IRF6 mRNA expression (*p* < 0.05, Fig. 1D,E). These findings demonstrate that IRF6 is a PPARγ co-regulator that inhibits PPARγ activity in cerebrovascular endothelial cells.

**Figure 2:**
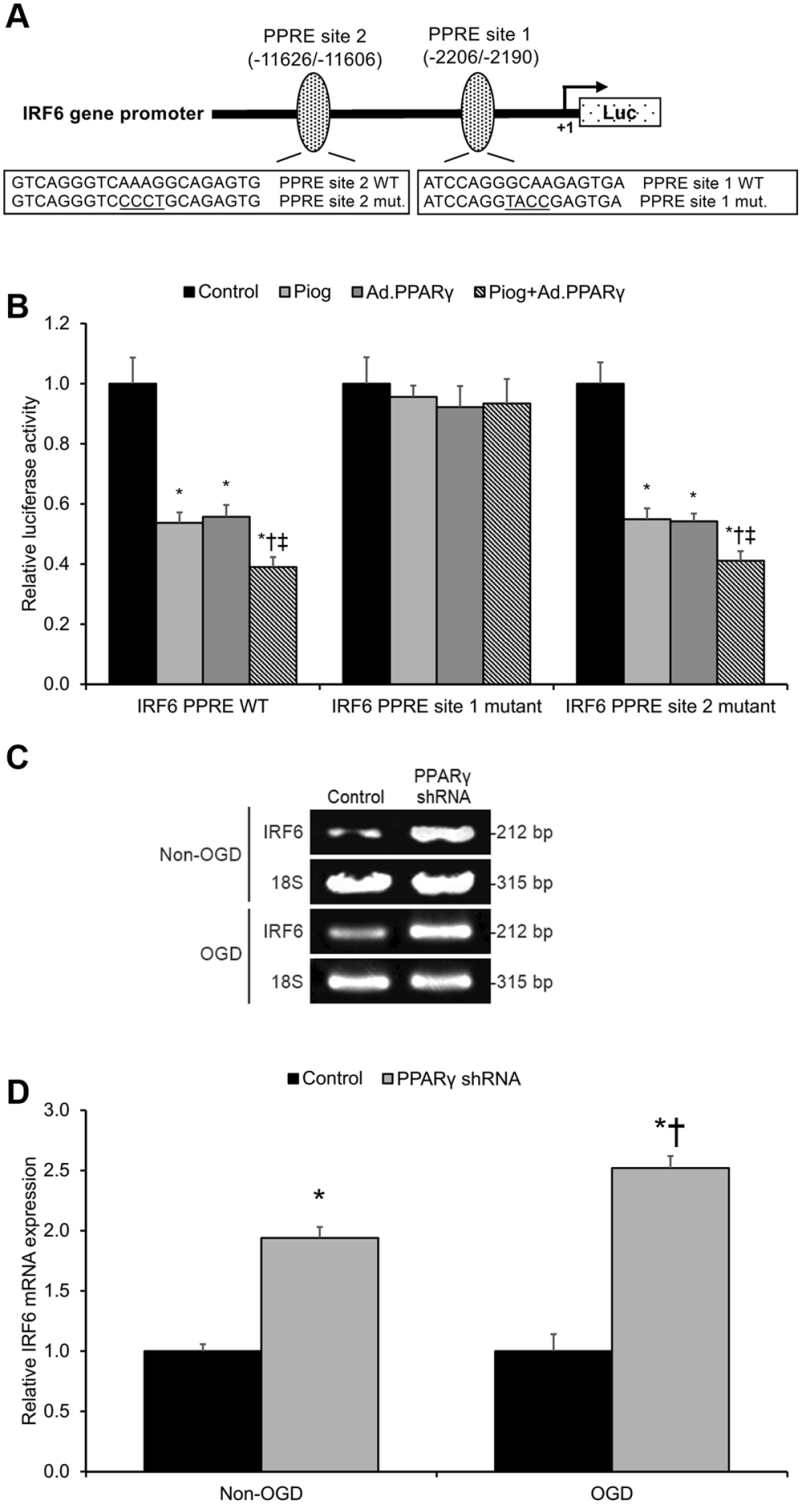
PPARγ Trans-Represses IRF6 in Cerebrovascular Endothelial Cells. (**A**) Diagram of the pGL 4.10 luciferase reporter plasmid carrying the murine IRF6 gene promoter sequence 5′-upstream of Luc. Two putative PPRE binding sites were identified at −2206/−2190 bp (PPRE site 1) and −11626/−11606 bp (PPRE site 2). Transcription starts at +1 bp. (**B**) A luciferase reporter assay was conducted by transfecting murine cerebrovascular endothelial cells with the pGL 4.10 luciferase reporter plasmid delivering either the IRF6 wild-type promoter sequence (IRF6 PPRE WT) or the IRF6 promoter sequence carrying a mutation at one of the two PPRE sites (IRF6 PPRE site 1 mutant or IRF6 PPRE site 2 mutant). Pioglitazone and/or PPARγ gain-of-function significantly elevated IRF6 wild-type promoter activity but did not affect IRF6 PPRE site 1 mutant promoter activity. *P < 0.05 versus control group, ^†^ *P* < 0.05 versus Pioglitazone group, ^‡^ *P* < 0.05 versus Ad.PPARγ group. (**C,D**) Real-time PCR on the total RNA content from control and small-hairpin PPARγ RNA (PPARγ shRNA)-transfected murine cerebrovascular endothelial cells revealed that PPARγ silencing significantly elevates IRF6 mRNA expression, particularly more significantly under oxygen-glucose deprivation (OGD) conditions. Cropped blots are displayed here. *P < 0.05 versus same-condition Control group, ^†^ *P* < 0.05 versus Non-OGD PPARγ shRNA group. Data are reported as means ± standard errors of the mean (SEMs).

To further investigate pioglitazone-driven inhibition of IRF6 transcription in mouse cerebrovascular endothelial cells, we searched for the presence of potential PPRE sites (PPAR binding sites) in the murine IRF6 promoter region. Two putative PPRE sites were localized to the murine IRF6 gene promoter: −2206/−2190 bp (PPRE site 1) and −11626/−11606 bp (PPRE site 2) (Fig. 2A). To assess the function of these two PPRE sites in the murine IRF6 gene promoter, a murine IRF6 promoter segment was cloned into a luciferase reporter vector, and a luciferase transcriptional assay was applied to assess PPARγ’s effect upon the transcriptional activity of the IRF6 promoter. Briefly, cerebrovascular endothelial cells were transfected with either the wild-type IRF6 promoter segment (IRF6 PPRE WT), a site-directed mutated IRF6 promoter with a mutated PPRE site 1 (IRF6 PPRE site 1 mutant), or a site-directed mutated IRF6 promoter with a mutated PPRE site 2 (IRF6 PPRE site 2 mutant). With or without pretreatment with pioglitazone, the cerebrovascular endothelial cells were co-infected with adenoviral GFP or adenoviral PPARγ. As a result, PPARγ overexpression and/or pioglitazone significantly inhibited IRF6 promoter-driven luciferase reporter activity (*p* < 0.05, Fig. 2B). Furthermore, luciferase activity driven by the IRF6 PPRE site 1 mutant (as opposed to the IRF6 PPRE site 2 mutant) failed to respond to PPARγ overexpression or pioglitazone (*p* > 0.05, Fig. 2B), demonstrating that the PPRE site 1 on the IRF6 promoter region is the binding site responsible for PPARγ’s inhibition of IRF6 mRNA expression in cerebrovascular endothelial cells. Consistent with the foregoing results, PPARγ silencing significantly promoted IRF6 mRNA expression in cerebrovascular endothelial cells under both non-OGD and OGD conditions (*p* < 0.05, Fig. 2C,D).

### **I**RF6 Silencing Promotes Pioglitazone-Mediated Cerebrovascular Protection against Ischemic Injury

**Figure 3:**
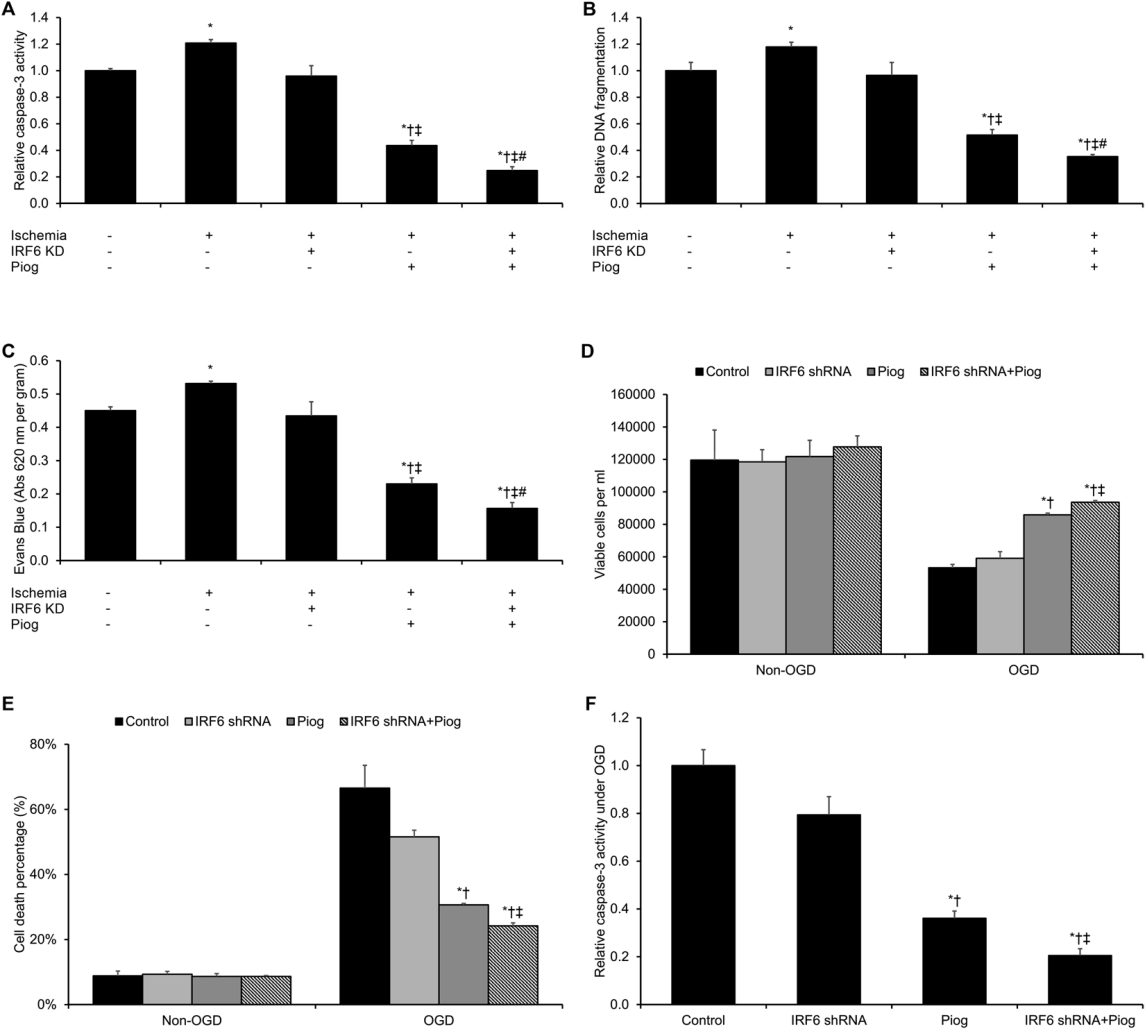
IRF6 Knockdown Potentiates Pioglitazone-Driven Cytoprotection in Cerebrovascular Endothelial Cells Following Ischemic Insult. (**A–C**) Wild-type mice or adenoviral-mediated IRF6 knockdown mice were treated with pioglitazone following middle cerebral artery (MCA) occlusion. After 24 hours of MCA reperfusion, cerebral microvessels were isolated and assessed for (**A**) caspase-3 activity and (**B**) DNA fragmentation. (**C**) Cerebrovascular permeability by Evans Blue extravasation was also assessed following 24 hours of MCA reperfusion. Pioglitazone significantly reduced caspase-3 activation, DNA fragmentation, and cerebrovascular permeability in wild-type mice. Notably, pioglitazone-driven vasoprotection was potentiated in IRF6 knockdown mice. *P < 0.05 versus sham group, ^†^ *P* < 0.05 versus Ischemia group, ^‡^ *P* < 0.05 versus Ischemia + IRF6 KD group, ^#^ *P* < 0.05 versus Ischemia + Piog group. (**D–F**) Control and IRF6 shRNA murine cerebrovascular endothelial cells were pre-treated with pioglitazone prior to oxygen-glucose deprivation (OGD) exposure. We assayed (**D**) cell viability by MTT assay, (**E**) cell death by the lactate dehydrogenase (LDH) kmethod, and (**F**) caspase-3 activity. IRF6 silencing potentiated pioglitazone-driven cytoprotection under *in vitro* OGD conditions. *P < 0.05 versus same-condition Control group, ^†^ *P* < 0.05 versus same-condition IRF6 shRNA group, ^‡^ *P* < 0.05 versus same-condition Piog group. Data are reported as means ± standard errors of the mean (SEMs).

In order to investigate IRF6’s role (if any) in pioglitazone-driven cerebrovascular endothelial protection *in vivo*, IRF6 knockdown and WT mice treated with pioglitazone or vehicle were subjected to MCA occlusion. Pioglitazone significantly reduced ischemia-driven caspase-3 activity (*p* < 0.05, Fig. 3A), significantly reduced DNA fragmentation (*p* < 0.05, Fig. 3B), and significantly ameliorated cerebrovascular permeability (*p* < 0.05, Fig. 3C) in WT cerebral microvasculature. Notably, these vasoprotective effects by pioglitazone were all significantly enhanced in IRF6 knockdown mice (all *p* < 0.05, Fig. 3A–C).

In order to validate IRF6’s role in pioglitazone-driven cerebrovascular endothelial protection *in vitro*, control and IRF6-silenced cerebrovascular endothelial cells were subjected to non-OGD and OGD conditions with or without pretreatment with pioglitazone. As a result, pioglitazone significantly promoted cell survival in OGD cerebrovascular endothelial cells (*p* < 0.05, Fig. 3D). Consistent with this result, pioglitazone significantly reduced caspase-3 activity under OGD conditions (*p* < 0.05, Fig. 3F). Notably, IRF6 silencing significantly enhanced pioglitazone cytoprotection (*p* < 0.05, Fig. 3D,E) and significantly reduced OGD-driven caspase-3 activity (*p* < 0.05, Fig. 3F) in cerebrovascular endothelial cells under OGD conditions. These findings suggest that IRF6 is a key PPARγ co-regulator in pioglitazone-driven cerebrovascular endothelial protection under ischemic conditions.

### miR-106a is Upregulated in Ischemic Cerebrovascular Endothelial Cells

**Figure 4:**
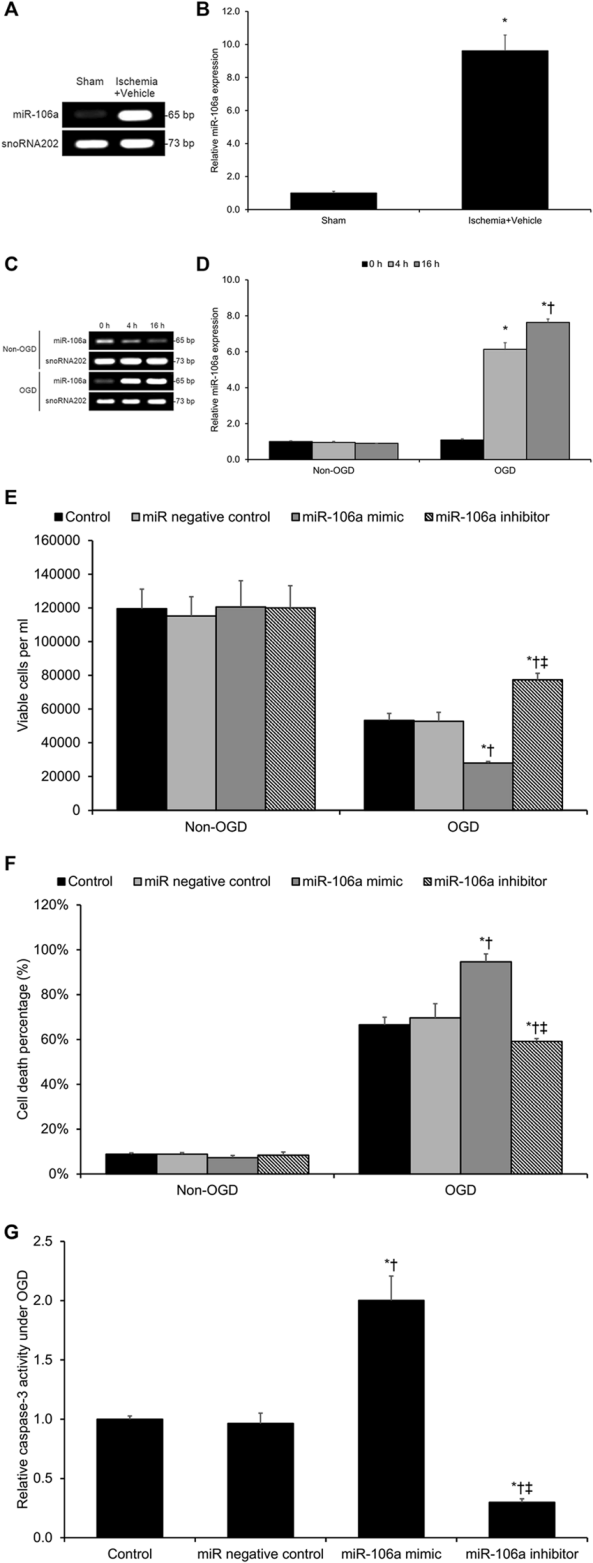
Ischemia-Induced miR-106a Upregulation Promotes OGD-Driven Cerebrovascular Endothelial Cell Death. (**A,B**) Real-time PCR on the total RNA content from cerebral microvessels extracted from wild-type mouse brains following middle cerebral artery (MCA) occlusion revealed cerebral ischemia-induced miR-106a upregulation. Cropped blots are displayed here. *P < 0.05 versus sham group. (**C,D**) Real-time PCR on the total RNA content from murine cerebrovascular endothelial cells subjected to oxygen-glucose deprivation (OGD) exposure revealed OGD exposure time-dependent miR-106a upregulation. Cropped blots are displayed here. *P < 0.05 versus same-condition 0 h group, ^†^ *P* < 0.05 versus same-condition 4 h group. (**E–G**) Murine cerebrovascular endothelial cells were pre-treated with either a miR-106a mimic or a miR-106a inhibitor prior to OGD exposure. We assayed (**E**) cell viability by MTT assay, (**F**) cell death by the lactate dehydrogenase (LDH) method, and (**G**) caspase-3 activity. The miR-106a mimic significantly promoted OGD-driven cell death and caspase-3 activity, while the miR-106a inhibitor produced the opposite effects. Treatment with the scrambled-sequence microRNA negative control produced no significant effect. *P < 0.05 versus same-condition Control group, ^†^ *P* < 0.05 versus same-condition miR negative control group, ^‡^ *P* < 0.05 versus same-condition miR-106a mimic group. Data are reported as means ± standard errors of the mean (SEMs).

Yin *et al*.’s previous work has demonstrated that pioglitazone exerts its cerebrovascular protective effect through a miR-15a-associated mechanism^17^. Another microRNA–miR-106a–has been shown to be upregulated in animal models of retinal and renal ischemia^20, 21^, and the miR-106a promoter has been shown to be enriched for IRF6 binding sites^22^. On the basis of this previous evidence, we hypothesized that miR-106a may be upregulated in ischemic cerebrovascular endothelial cells. To test this hypothesis, we constructed an *in vivo* murine model of transient cerebral ischemia via transient MCA occlusion and assessed miR-106a expression 24 hours post-MCA occlusion. Transient MCA occlusion significantly promoted *in vivo* miR-106a expression (*p* < 0.05, Fig. 4A,B). Similar to these *in vivo* findings, OGD significantly promoted *in vitro* miR-106a expression in cerebrovascular endothelial cell cultures in a time-dependent manner (*p* < 0.05, Fig. 4C,D). These findings show that ischemia increases the expression of miR-106a.

### miR-106a Promotes OGD-Driven Cell Death in Cerebrovascular Endothelial Cells

As miR-106a is upregulated in cerebrovascular endothelial cells in response to ischemia, we hypothesized that miR-106a may serve a pro-apoptotic function in these cells under ischemic conditions. To test this hypothesis, gain-of-miR-106a function or loss-of-miR-106a function was produced in cerebrovascular endothelial cells through transfection of a miR-106a mimic or a miR-106a inhibitor, respectively. Application of the miR-106a mimic produced significant decreases in cerebrovascular endothelial cell viability and significant increases in cerebrovascular endothelial cell death after 16 hours under OGD conditions (*p* < 0.05, Fig. 4E,F). Consistently, application of the miR-106a inhibitor significantly increased OGD-induced cerebrovascular endothelial cell viability and significantly reduced OGD-induced cerebrovascular endothelial cell death (*p* < 0.05, Fig. 4E,F). Moreover, the miR-106a mimic significantly promoted OGD-induced caspase-3 activity, while the miR-106a inhibitor significantly reduced OGD-induced caspase-3 activity (*p* < 0.05, Fig. 4G). These findings show that miR-106a induces cerebrovascular endothelial cell death under OGD conditions.

### PPARγ Activation Inhibits Pro-Apoptotic miR-106a Expression in OGD Cerebrovascular Endothelial Cells

**Figure 5:**
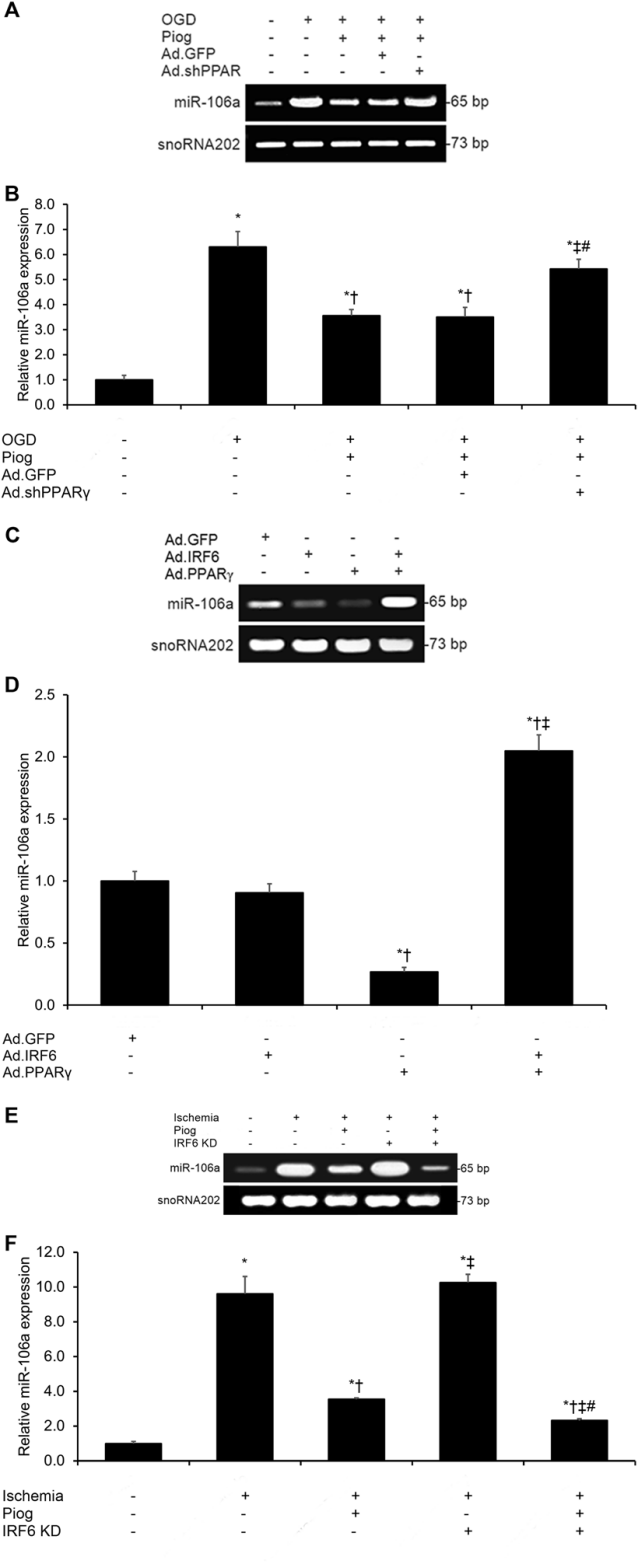
IRF6 Inhibits PPARγ-Induced Repression of miR-106a Expression in Ischemic Cerebrovascular Endothelial Cells. (**A,B**) Control GFP and small-hairpin PPARγ RNA (PPARγ shRNA)-transfected murine cerebrovascular endothelial cells were pre-treated with pioglitazone prior to oxygen-glucose deprivation (OGD) exposure. Real-time PCR on the total RNA content from these cells revealed that OGD-induced miR-106a upregulation was reversed by pioglitazone, and this effect was dependent upon PPARγ. Cropped blots are displayed here. *P < 0.05 versus sham group, ^†^ *P* < 0.05 versus OGD group, ^‡^ *P* < 0.05 versus OGD + Piog group, ^#^ *P* < 0.05 versus OGD + Piog + Ad.GFP group. (**C,D**) Real-time PCR on the total RNA content from murine cerebrovascular endothelial cells transfected with adenoviral GFP, IRF6, PPARγ, or IRF6 + PPARγ revealed that PPARγ gain-of-function significantly reduced miR-106a expression. IRF6 gain-of-function alone had no significant impact upon miR-106a expression, but it significantly inhibited PPARγ suppression of miR-106a expression. Cropped blots are displayed here. *P < 0.05 versus Ad.GFP group, ^†^ *P* < 0.05 versus Ad.IRF6 group, ^‡^ *P* < 0.05 versus Ad.PPARγ group. (**E,F**) Wild-type mice or adenoviral-mediated IRF6 knockdown mice were treated with pioglitazone following middle cerebral artery (MCA) occlusion. After 24 hours of MCA reperfusion, cerebral microvessels were isolated. Real-time PCR on the total RNA content from these cerebral microvessels revealed that cerebral ischemia-induced miR-106a upregulation was reversed by pioglitazone. IRF6 knockdown alone had no significant impact upon miR-106a transcription, but IRF6 knockdown significantly promoted pioglitazone-driven PPARγ suppression of miR-106a transcription. Cropped blots are displayed here. *P < 0.05 versus sham group, ^†^ *P* < 0.05 versus Ischemia group, ^‡^ *P* < 0.05 versus Ischemia + Piog group, ^#^ *P* < 0.05 versus Ischemia + IRF6 KD group.

On the basis of this previous evidence, we hypothesized that miR-106a may be a potential downstream target of pioglitazone suppression. We found that OGD-induced miR-106a upregulation was substantially reversed after pioglitazone treatment (*p* < 0.05, Fig. 5A,B). Notably, pioglitazone’s inhibition of miR-106a expression was significantly reversed by PPARγ silencing in cerebrovascular endothelial cells (*p* < 0.05, Fig. 5A,B). These findings indicate that miR-106a is a downstream target of pioglitazone suppression in cerebrovascular endothelial cells under OGD conditions.

### IRF6 Inhibits PPARγ-Induced Repression of miR-106a Expression in Ischemic Cerebrovascular Endothelial Cells

As we have already demonstrated that IRF6 is a key PPARγ co-regulator in pioglitazone-driven cerebrovascular endothelial protection under ischemic conditions, we next hypothesized that IRF6 may negatively affect PPARγ’s repression of miR-106a expression in cerebrovascular endothelial cells. Consistent with this hypothesis, we found that adenoviral-driven PPARγ overexpression significantly inhibited miR-106a expression in cerebrovascular endothelial cells *in vitro* (*p* < 0.05, Fig. 5C,D), while adenoviral-driven IRF6 overexpression significantly reversed this effect (*p* < 0.05, Fig. 5C,D).

To test further this hypothesis *in vivo*, we constructed an *in vivo* murine model of transient cerebral ischemia via MCA occlusion in WT and IRF6 knockdown mice and assessed pioglitazone’s effects upon miR-106a expression 24 hours post-MCA occlusion. Transient MCA occlusion-induced miR-106a upregulation was substantially reversed after pioglitazone treatment (*p* < 0.05, Fig. 5E,F). Notably, this suppression of miR-106a expression by pioglitazone was significantly enhanced in IRF6 knockdown mice (*p* < 0.05, Fig. 5E,F). These findings indicate that IRF6 inhibits PPARγ’s repression of miR-106a expression in ischemic cerebrovascular endothelial cells.

### IRF6 Interferes with PPARγ’s Suppressive Binding of the miR-106a Promoter in Cerebrovascular Endothelial Cells

**Figure 6:**
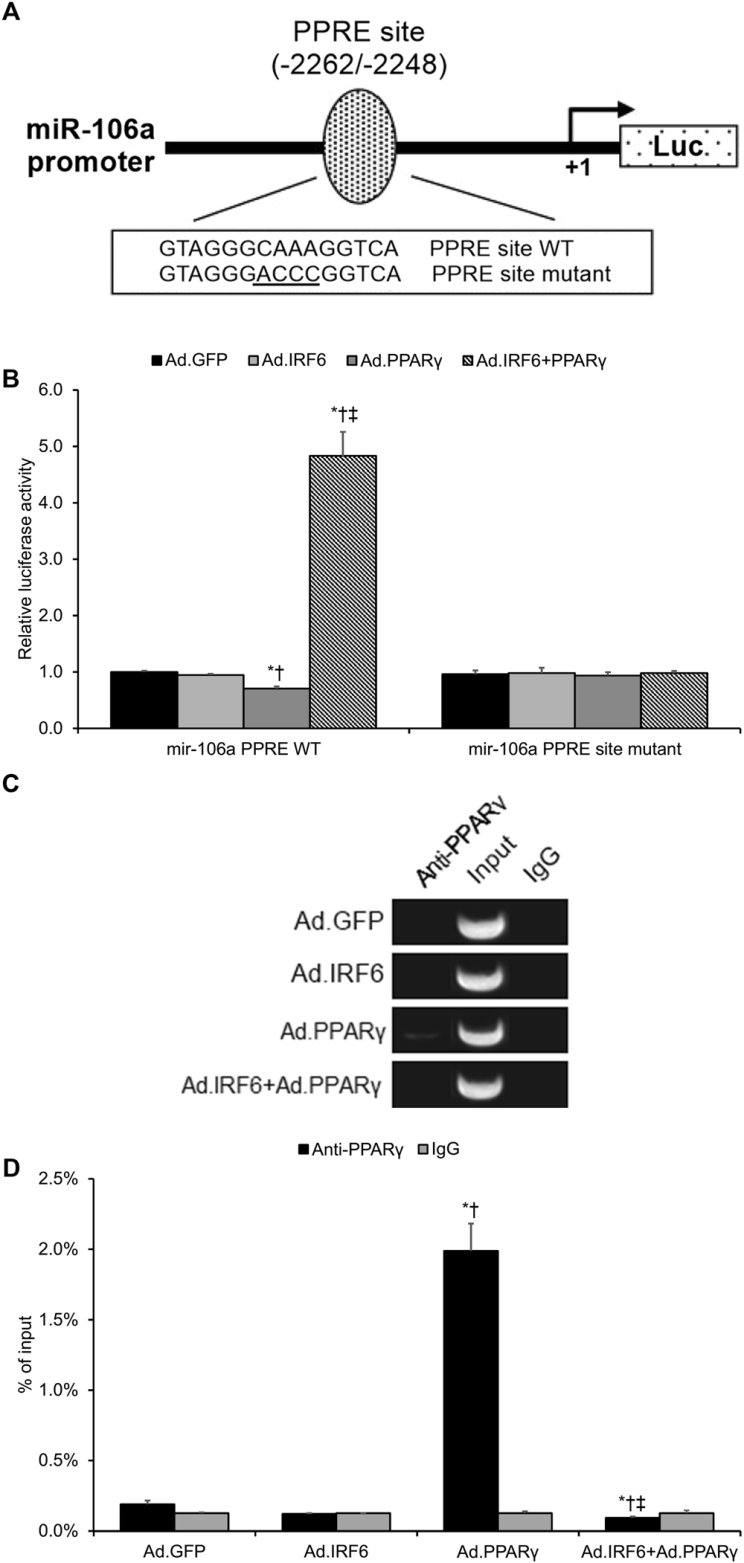
IRF6 Interferes with PPARγ’s Suppressive Binding of the miR-106a Promoter in Cerebrovascular Endothelial Cells. (**A**) Diagram of the pGL 4.10 luciferase reporter plasmid carrying the murine miR-106a gene promoter sequence 5′-upstream of Luc. A putative PPRE binding site was identified at −2262/−2248 bp (PPRE site). Transcription starts at +1 bp. (**B**) A luciferase reporter assay was conducted by transfecting murine cerebrovascular endothelial cells with the pGL 4.10 luciferase reporter plasmid delivering either the miR-106a wild-type promoter sequence (miR-106a PPRE WT) or the miR-106a promoter sequence carrying a mutation at the PPRE site (miR-106a PPRE site mutant). PPARγ gain-of-function significantly reduced miR-106a wild-type promoter activity but did not significantly affect miR-106a PPRE site mutant promoter activity. IRF6 gain-of-function alone had no significant impact upon miR-106a transcription, but it significantly inhibited PPARγ suppression of miR-106a transcription. *P < 0.05 versus same-condition Ad.GFP group, ^†^ *P* < 0.05 versus same-condition Ad.IRF6 group, ^‡^ *P* < 0.05 versus same-condition Ad.PPARγ group. (**C**) PCR products from the chromatin immunoprecipitation (ChIP) assay using miR-106a promoter-specific PCR primers and anti-PPARγ antibodies. Cropped blots are displayed here. (**D**) Real-time PCR data from ChIP assay with the appropriate controls. *P < 0.05 versus Ad.GFP group, ^†^ *P* < 0.05 versus Ad.IRF6 group, ^‡^ *P* < 0.05 versus Ad.PPARγ group. Data are reported as means ± standard errors of the mean (SEMs).

As miR-106a shows evidence of being a downstream target of pioglitazone suppression, we hypothesized that the promoter region of miR-106a may possess a PPRE site. Consistent with our hypothesis, we localized a putative PPRE site in the murine miR-106a promoter at −2262 to −2248 bp (Fig. 6A). We then hypothesized that PPARγ may bind to this putative PPRE site and suppress miR-106a transcription and that IRF6 may act to interfere with this transcriptional suppression. We transfected cerebrovascular endothelial cells with a luciferase reporter vector containing a miR-106a promoter segment with either the wild-type PPRE site or a mutated PPRE site. The cerebrovascular endothelial cells were then co-infected with adenoviral PPARγ, IRF6, PPARγ + IRF6, or GFP. As a result, we found that adenoviral-driven PPARγ overexpression significantly inhibited miR-106a transcriptional activity (*p* < 0.05, Fig. 6B). Moreover, adenoviral-driven IRF6 overexpression alone had no significant effect upon miR-106a transcriptional activity (*p* > 0.05, Fig. 6B); however, adenoviral-driven IRF6 overexpression in concert with PPARγ overexpression did significantly inhibit PPARγ suppression of miR-106a transcriptional activity (*p* < 0.05, Fig. 6B). Notably, luciferase activity from the mutated PPRE site was not significantly affected by adenoviral-driven PPARγ and/or IRF6 overexpression (*p* > 0.05, Fig. 6B), demonstrating that the PPRE site in question is responsible for PPARγ’s repression of miR-106a transcription. Moreover, ChIP analysis revealed that PPARγ binds to the miR-106a promoter and that this binding is perturbed by IRF6 overexpression (*p* < 0.05, Fig. 6C,D). In sum, these findings suggest that IRF6 acts as a PPARγ co-repressor and interferes with PPARγ’s suppressive binding of the miR-106a promoter.

## Discussion

Here, we demonstrated that IRF6 directly binds to PPARγ and inhibits PPARγ activity in murine cerebrovascular endothelial cells. We also found that silencing of IRF6 expression promotes pioglitazone-driven cytoprotection in murine cerebrovascular endothelial cells under ischemic conditions. Finally, we demonstrated that miR-106a is upregulated in murine cerebrovascular endothelial cells under ischemic conditions and is a direct target of the PPARγ/IRF6 complex. This study is the first to report that IRF6 is a novel PPARγ co-suppressor that suppresses PPARγ-mediated cerebrovascular endothelial cytoprotection following ischemia.

The nuclear receptor PPARγ is strongly expressed in the cerebrovasculature^23, 24^, and PPARγ-based cerebrovascular protection following ischemic assault has been extensively demonstrated in animal stroke models and human stroke patients^25^. On this basis, PPARγ shows promise as a potential pharmacotherapeutic target for stroke patients. For example, the synthetic PPARγ-activating compounds termed thiazolidinediones (e.g., pioglitazone, rosiglitazone) show evidence of ameliorating cerebral damage and neurological outcomes in animal models of cerebral ischemia as well as lowering recurrent stroke risk in stroke patients^26–28^. Recently, Yin *et al*. has demonstrated that the PPARγ activator pioglitazone ameliorates ischemia-induced cerebrovascular endothelial cell death both *in vitro* and *in vivo*, and adenoviral-driven PPARγ gain-of-function bolsters this pioglitazone-driven cytoprotection in cultured murine cerebrovascular endothelial cells^9^. Our current findings support those of Yin *et al*., as we also demonstrated that pioglitazone-driven PPARγ activation protects murine cerebrovascular endothelial cells under ischemic conditions.

Previous research has established that the functioning of nuclear receptors–such as PPARγ, liver X receptor (LXR), and retinoic acid receptor (RAR) – is heavily dependent upon a network of co-regulators that serve to either co-activate or co-repress the regulatory activity of the nuclear receptor^29^. Notably, accumulated research on nuclear receptor co-regulation has revealed that co-repressors can be actively exchanged with co-activators, a phenomenon termed ‘de-repression^30^’. For example, in adipocytes, treatment with thiazolidinedione has been demonstrated to release the PPARγ co-repressor histone deacetylase complex (HDAC) while simultaneously recruiting PPARγ co-activators to the PPRE site on the glycerol kinase (GyK) gene, thereby ‘de-repressing’ GyK transcription^31^. Here, we demonstrated that IRF6 is a PPARγ co-repressor that inhibits PPARγ transcriptional activity in murine cerebrovascular endothelial cells. Notably, we also found that IRF6 activity was subject to PPARγ trans-repression, suggesting a PPARγ-IRF6 regulatory feedback loop in cerebrovascular endothelial cells. Moreover, IRF6 silencing served to promote the PPARγ-driven reduction in cerebrovascular endothelial cell death under ischemic conditions. As Yin *et al*. recently demonstrated that KLF11 is a PPARγ co-activator that promotes PPARγ transcriptional activity in murine cerebrovascular endothelial cells under identical experimental conditions^9^, our combined findings suggest that pioglitazone treatment may serve to ‘de-repress’ PPARγ transcriptional activity by triggering the exchange of the PPARγ co-repressor IRF6 with the PPARγ co-activator KLF11.

As Yin *et al*. demonstrated that the transcription factor KLF11 synergistically enhances PPARγ-based suppression of pro-apoptotic miR-15a expression in cerebrovascular endothelial cells^9^, we hypothesized that IRF6’s co-repression of PPARγ-driven vasculoprotection may involve the regulation of endothelial cell apoptosis via a microRNA-based mechanism. Previous research reveals that miR-106a is upregulated in animal models of retinal and renal ischemia^20, 21^, and the miR-106a promoter has been shown to be enriched for IRF6 binding sites^22^. On this basis, we hypothesized that miR-106a may serve a pro-apoptotic function under ischemic conditions and may be a potential downstream target of pioglitazone suppression in murine cerebrovascular endothelial cells. Consistent with our hypothesis, *in vitro* OGD and *in vivo* MCA occlusion produced significant miR-106a upregulation in cerebrovascular endothelial cells and the cerebral microvasculature, respectively. Moreover, application of a miR-106a mimic induces cerebrovascular endothelial cell death under OGD conditions. Pioglitazone significantly inhibited pro-apoptotic miR-106a expression in a PPARγ-dependent manner.

As miR-106a shows evidence of being a downstream target of pioglitazone suppression, we next hypothesized that the promoter region of miR-106a may possess a PPRE site and that IRF6 may act to modulate PPARγ transcriptional suppression of miR-106a expression in murine cerebrovascular endothelial cells. Applying a functional analysis of the PPRE site on the miR-106a promoter, we found that PPARγ directly suppresses miR-106a transcription. Moreover, we found that IRF6 inhibits PPARγ’s suppression of miR-106a expression, and our associated ChIP analysis demonstrated that IRF6 inhibits PPARγ’s association with its response element on the miR-106a promoter. These results suggest that IRF6 may promote pro-apoptotic miR-106a expression via inhibiting PPARγ suppression of miR-106a-associated mechanism(s), thereby weakening the vascular endothelium against ischemic damage.

The favorable results of thiazolidinedione use in animal models of stroke suggest that these drugs may be useful therapeutic tools for stroke patients^25^. Indeed, clinical trials have revealed that pioglitazone reduces recurrent stroke risk in stroke patients^27, 28^. On this basis, Yin *et al*. has suggested that improving the identification and characterization of PPARγ co-regulators should result in superior PPARγ-based therapeutic agents^9^. This study has identified IRF6’s role as a novel PPARγ co-suppressor that suppresses PPARγ-mediated cerebrovascular endothelial cytoprotection following ischemia. The current findings should aid future researchers in better characterizing IRF6 as a novel therapeutic target for modulating PPARγ -associated signaling cascades for purposes of stroke treatment.

## Electronic supplementary material

Dataset 1
