# Supplementary material for: The Transcription Factor IRF6 Co-Represses PPARγ-Mediated Cytoprotection in Ischemic Cerebrovascular Endothelial Cells
Source: Sci Rep. 2017 May 19;7:2150. doi: 10.1038/s41598-017-02095-3 (PMC5438409; doi:10.1038/s41598-017-02095-3)

**The Transcription Factor IRF6 Co-Represses PPARγ-Mediated Cytoprotection in**

**Ischemic Cerebrovascular Endothelial Cells**

**Running title: IRF6 Co-Represses PPARγ in Ischemic Cerebrovascular Endothelium**

Rongzhong Huang^1#^, Zicheng Hu^2#^, Yuxing Feng^3^, Lehua Yu^1^, and Xingsheng Li^4^

1. Department of Rehabilitation Medicine, the Second Affiliated Hospital of Chongqing Medical University, Chongqing, China
2. Department of Neurology, Institute of Surgery Research, Daping Hospital, Third Military Medical

University, Chongqing, China

1. Department of Neurology, the Ninth People’s Hospital of Chongqing, Chongqing, China
2. Department of Gerontology, the Second Affiliated Hospital of Chongqing Medical University, Chongqing, China

^#^Co-first authors

**SUPPLEMENTARY FIGURE LEGENDS**

**Supplementary Figure 1. Validation of Adenoviral-Mediated IRF6 Knockdown in Murine Cerebral Vasculature**

Western blotting showing downregulation of IRF6 protein expression in isolated cerebral microvessels following in vivo adenoviral knockdown. Cropped blots are displayed here.


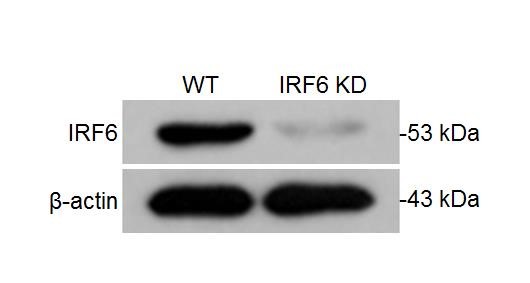


**Supplementary Figure 2. Validation of Adenoviral-Mediated Gain-of-Function or Loss-of-**

**Function in Cultured Cerebrovascular Endothelial Cells**

(A) Western blotting showing successful upregulation or downregulation of PPARγ protein expression following in vitro adenoviral infection. Cropped blots are displayed here. (B) Western blotting showing upregulation or downregulation of IRF6 protein expression following in vitro adenoviral infection. Cropped blots are displayed here. (C) Western blotting showing upregulation of both PPARγ and IRF6 following dual in vitro adenoviral infection. Cropped blots are displayed here.


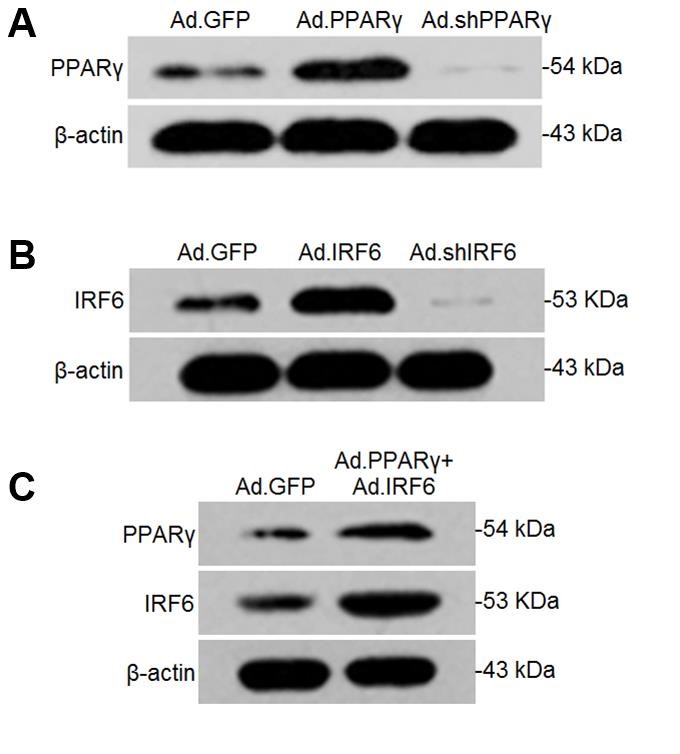

Supplement: Supplementary file 1 — Dataset 1 [file 41598_2017_2095_MOESM1_ESM.doc]
